# Bifunctional Enzyme SpoT Is Involved in Biofilm Formation of Helicobacter pylori with Multidrug Resistance by Upregulating Efflux Pump Hp1174 (*gluP*)

**DOI:** 10.1128/AAC.00957-18

**Published:** 2018-10-24

**Authors:** Xiaoran Ge, Yuying Cai, Zhenghong Chen, Sizhe Gao, Xiwen Geng, Ya Li, Yan Li, Jihui Jia, Yundong Sun

**Affiliations:** a Department of Microbiology, Key Laboratory for Experimental Teratology of Ministry of Education, Shandong Provincial Key Laboratory of Infection and Immunology, Shandong University School of Basic Medicine, Jinan, Shandong, People's Republic of China; b Department of Microbiology, Guiyang Medical University, Guiyang, Guizhou, People's Republic of China; c School of Control Science and Engineering, Shandong University, Jinan, People's Republic of China

**Keywords:** Helicobacter pylori, SpoT, efflux pump, biofilm, antibiotic resistance, GluP

## Abstract

The drug resistance of Helicobacter pylori is gradually becoming a serious problem. Biofilm formation is an important factor that leads to multidrug resistance (MDR) in bacteria. The ability of H. pylori to form biofilms on the gastric mucosa is known. However, there are few studies on the regulatory mechanisms of H. pylori biofilm formation and multidrug resistance. Guanosine 3′-diphosphate 5′-triphosphate and guanosine 3′,5′-bispyrophosphate [(p)ppGpp] are global regulatory factors and are synthesized in H. pylori by the bifunctional enzyme SpoT. It has been reported that (p)ppGpp is involved in the biofilm formation and multidrug resistance of various bacteria. In this study, we found that SpoT also plays an important role in H. pylori biofilm formation and multidrug resistance. Therefore, it was necessary to carry out some further studies regarding its regulatory mechanism. Considering that efflux pumps are of great importance in the biofilm formation and multidrug resistance of bacteria, we tried to determine whether efflux pumps controlled by SpoT participate in these activities. We found that Hp1174 (glucose/galactose transporter [*gluP*]), an efflux pump of the major facilitator superfamily (MFS), is highly expressed in biofilm-forming and multidrug-resistant (MDR) H. pylori strains and is upregulated by SpoT. Through further research, we determined that *gluP* is involved in H. pylori biofilm formation and multidrug resistance. Furthermore, the average expression level of *gluP* in the clinical MDR strains (C-MDR) was considerably higher than that in the clinical drug-sensitive strains (C-DSS). Taken together, our results revealed a novel molecular mechanism of H. pylori resistance to multidrug exposure.

## Abstract (precis)

The drug resistance of Helicobacter pylori is gradually becoming a serious problem. Biofilm formation is an important factor that leads to multidrug resistance (MDR) in bacteria.

## INTRODUCTION

Helicobacter pylori, the rate of infection with which is over 50% throughout the world, is highly associated with a wide range of upper gastrointestinal diseases, especially gastric carcinoma (1). Contemporaneously, the most common method of treatment of infections caused by this bacterium is called triple therapy, which consists of a proton pump inhibitor and two antibiotics, namely, macrolides, nitroimidazoles, or β-lactams (2). However, in recent years, eradication of H. pylori has become increasingly difficult because the rate of antibiotic resistance acquisition by H. pylori has generally increased (3). In addition, with the extended use of antibiotics, the appearance of multidrug-resistant (MDR) H. pylori strains, which are resistant to multiple antibiotics, has been reported (4).

There are numerous molecular mechanisms that contribute to multidrug resistance in the bacteria, including decreased drug permeation, efflux pumps, alteration or bypass of the drug target, production of antibiotic-modifying enzymes, and other physiological states, such as the formation of biofilms (5). Biofilms are communities of microorganisms that are anchored to a surface and live in an extracellular matrix made up of extracellular polymeric substances (EPS); this matrix is produced by the microorganisms to form their immediate environment (6). The property that makes biofilms distinct from planktonic cells is their increased resistance to antimicrobial agents. It has recently become widely accepted that biofilms play an important role in the pathogenesis of some chronic human infections, as well as bacterial multidrug resistance (6, 7). Furthermore, H. pylori has the ability to form biofilms *in vitro* (8). In 2006, using scanning electron microscopy (SEM), Carron et al. first proposed that H. pylori could form biofilms *in vivo* (9). The formation of biofilms *in vivo* is an important cause of H. pylori resistance to multiple antibiotics (10), as is its stringent response to a stressful environment lacking nutrients (11).

The stringent response is a bacterial stress response that controls bacterial adaptation to stress signals, such as nutrient deprivation (12). In bacteria, the signal molecules guanosine 3′-diphosphate 5′-triphosphate and guanosine 3′,5′-bispyrophosphate [(p)ppGpp], which are induced by diverse stresses, activate the stringent response (12, 13). The phenomenon of (p)ppGpp affecting bacterial multidrug resistance has already been reported for some other bacteria (14). Maisonneuve et al. reported that under antibiotic stress, Escherichia coli can produce rare cells that transiently become multidrug tolerant (15). In these rare cells, the level of (p)ppGpp was high (15). In addition, it has also been proven that (p)ppGpp can affect the formation of the bacterial biofilm. For example, Sugisaki et al. determined that the accumulation of (p)ppGpp accelerated the formation of biofilms in Bordetella pertussis (16), and Li et al. reported that the low level of (p)ppGpp contributed to the formation of biofilms in Actinobacillus pleuropneumoniae S8 (17). Nevertheless, there are still no reports certifying the relationship between (p)ppGpp and the formation of biofilms in H. pylori.

In many bacteria, such as E. coli, (p)ppGpp is synthesized by two enzymes, RelA and SpoT (18), and SpoT is a bifunctional enzyme with both (p)ppGpp synthetase and hydrolase activity (18). However, there is only one member of the RelA/SpoT family, SpoT, in the H. pylori genome (19–21), and it is also a bifunctional enzyme (22); therefore, in H. pylori, we focused on whether SpoT is involved in H. pylori biofilm formation and multidrug resistance.

There are a series of transport proteins in bacteria that can acquire nutrients and extrude metabolic by-products, and some of these proteins are called efflux pumps (23). Efflux pumps have the ability to expel a broad range of antibiotics and have been recognized as significant components of multidrug resistance in many bacteria (24, 25), such as H. pylori (26–32). It has been demonstrated that efflux pumps work as one of the mechanisms that contribute to the antimicrobial resistance of biofilms (33), and evidence can be found in several microorganisms, such as Pseudomonas aeruginosa (34), E. coli (35), and Candida albicans (36). Moreover, Yonezawa et al. reported that in H. pylori clinical MDR strains (C-MDR), the expression of some efflux pump genes was elevated in biofilm cells compared to that in planktonic cells (37). While some efflux transporters have been detected in H. pylori 26695 (21), their functions, especially their functions in H. pylori biofilm formation, must be further studied.

Considering that SpoT is a global regulator, we suppose that SpoT can influence the formation of biofilms and multidrug resistance by regulating the expression of the efflux pump in H. pylori.

## RESULTS

### SpoT is involved in H. pylori biofilm formation and multidrug resistance.

**FIG 1:**
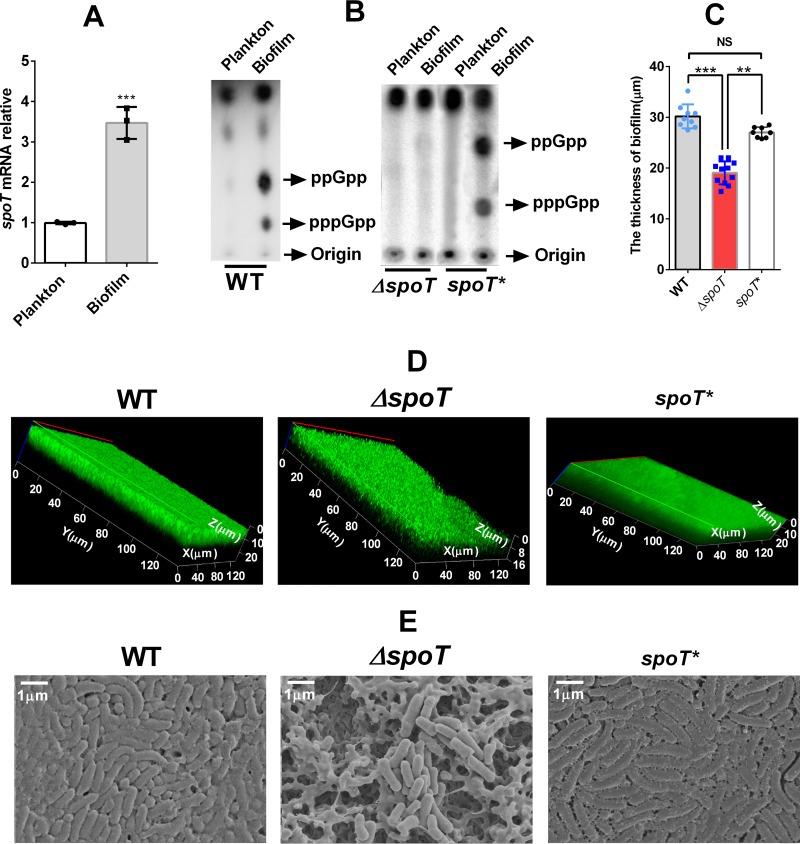
SpoT is involved in H. pylori biofilm formation. (A) mRNA expression of SpoT in biofilm-forming and planktonic cells determined by qRT-PCR. The levels of the signals were normalized to the 16S rRNA levels. Data are the means ± standard errors of the means from three independent experiments. Significance was determined by the unpaired Student's *t* test. ***, *P* < 0.001. (B) (p)ppGpp was induced in the biofilm-forming cells (of the wild-type [WT] and *spoT*-complemented [spoT*] strains) but not in the planktonic cells or the *spoT* mutant strain (the Δ*spoT* strain). ^32^P-labeled nucleotides of formic acid extracts of H. pylori were detected by thin-layer chromatography. Planktonic H. pylori bacteria were grown to the exponential phase. (C) Comparison of the biofilm thickness of the WT, Δ*spoT*, and spoT*strains, using data from the assay whose results are presented in panel D. The data presented are the means ± standard errors of the means from three independent experiments. Significance was determined by the paired Student's *t* test. **, *P* < 0.01; ***, *P* < 0.001; NS, not significant. (D) Confocal laser scanning microscopy (CLSM) images of WT, Δ*spoT*, and spoT* strain biofilms. Cells stained with membrane-permeant SYTO 9 (green) and membrane-impermeant propidium iodide (PI) (red) were visualized by confocal microscopy. (E) Scanning electron microscopy (SEM) images of WT, Δ*spoT*, and spoT*strain biofilms. The biofilm used in this experiment is a mature biofilm grown on a nitrocellulose membrane for 3 days, and the planktonic bacteria were from early exponential phase (OD_600_, approximately 0.4 to 0.5).

As a global regulatory factor, SpoT has been proven to participate in the formation of bacterial biofilms (16, 17, 38), while no related studies regarding H. pylori have been done. Therefore, we analyzed the difference in *spoT* expression between biofilm-forming and planktonic cells by real-time PCR (RT-PCR). The *spoT* gene is highly expressed in the latter (Fig. 1A). The (p)ppGpp production assay showed that the H. pylori cells in the biofilm produced more (p)ppGpp than did the planktonic cells (Fig. 1B). By constructing an *spoT* mutant strain (the Δ*spoT* strain) and an *spoT*-complemented strain (the spoT* strain), we compared the biofilms of the wild-type (WT), Δ*spoT*, and spoT* strains by confocal laser scanning microscopy (CLSM) (Fig. 1C and D) and SEM (Fig. 1E). LIVE/DEAD cell viability assays showed that the Δ*spoT* strain formed a lighter biofilm than did the WT strain and the spoT*strain (Fig. 1C and D). The WT strain and the spoT* strain could form complete and compact biofilms on the nitrocellulose (NC) membrane, whereas for the biofilm of the Δ*spoT* strain, the bacteria were not packed tightly enough, the biofilm matrix was not complete, and some cavities could be seen (Fig. 1E).

**FIG 2:**
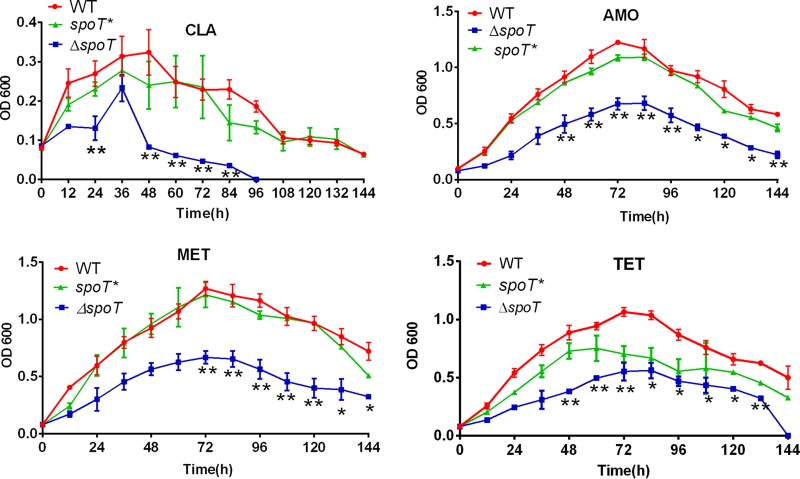
Comparison of growth inhibition curve characteristics of the WT, Δ*spoT*, and spoT*strains with various antibiotics (AMO [0.125 μg/ml], CLA [0.125 μg/ml], MET [0.5 μg/ml], TET [0.25 μg/ml]). Data are the means ± standard errors of the means from three independent experiments. The significance of the difference between the WT and Δ*spoT* strains was determined using the paired Student's *t* test. *, *P* < 0.05; **, *P* < 0.01.

According to the MIC of the WT strain and the Δ*spoT* strain, for planktonic cells, the Δ*spoT* strain was apparently more sensitive to various antibiotics, not including ciprofloxacin, than was the WT and spoT* strain (Table 1). With regard to the biofilm-forming cells, after knocking out *spoT*, cell resistance to various antibiotics, especially that to penicillin G, was reduced (Table 1). Then, we treated the WT, Δ*spoT*, and spoT* strains with antibiotics (clarithromycin [CLA], amoxicillin [AMO], tetracycline [TET], and metronidazole [MET]) at their MICs. The growth inhibitory curve demonstrated that the growth ability of the Δ*spoT* strain was obviously inhibited (Fig. 2).

**TABLE 1:** MICs determined for WT, MDR, Δ*spoT*, Δ*gluP*, spoT*, and gluP* strains in biofilm-forming and planktonic cells

Drug	MIC (μg/ml)											
	Planktonic cells							Biofilm-forming cells				
	WT	Δ*spoT*	spoT*	Δ*gluP*	gluP*	MDR-H	MDR-H (Δ*gluP*)	WT	Δ*spoT*	spoT*	Δ*gluP*	gluP*
Ampicillin (AMP)	0.0625	0.03125	0.0625	0.0156	0.0625	0.3125	0.156	3.75	0.9375	3.75	0.6	3.75
Penicillin G (PEN)	0.125	0.0156	0.0625	0.0078	0.0625	0.625	0.3125	2.5	0.234	2.5	0.078	1.25
Amoxicillin (AMO)	0.0625	0.0156	0.0625	0.0039	0.0625	0.8	0.3125	2.5	0.486	1.25	0.312	1.25
Clarithromycin (CLA)	0.125	0.0625	0.125	0.125	0.125	0.75	0.625	5	1.56	2.5	2.5	3.125
Tetracycline (TET)	0.25	0.0625	0.125	0.125	0.125	5	2.5	10	1.875	5	1.25	6.25
Ciprofloxacin (CIP)	0.25	0.25	0.25	0.25	0.25	4	1	5	2	5	3.75	5
Metronidazole (MET)	0.5	0.125	0.5	0.125	0.25	4	1.5	5	3.125	5	2.5	5
Furazolidone (FUR)	1	0.5	1	0.25	2	6	2	8	2.5	10	2	10

### Comparing the efflux capacity of the wild-type, Δ*spoT*, and spoT* strains.

**FIG 3:**
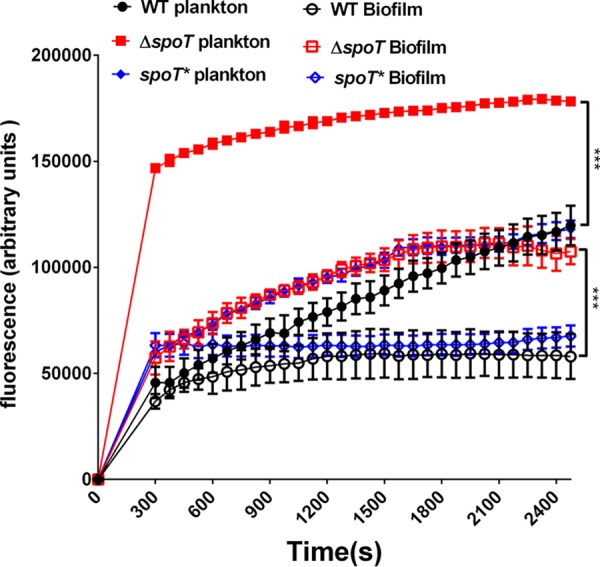
Comparison of the accumulation of Hoechst 33342 (2.5 M) in biofilm and planktonic cells of the WT, Δ*spoT*, and spoT*strains. The fluorescence intensity was recorded at excitation and emission wavelengths of 350 and 460 nm, respectively, over a 30-min incubation period. The data presented are the means ± standard errors of the means from three separate experiments. The paired Student's *t* test was performed to compare the accumulation of Hoechst 33342 between the WT and Δ*spoT* strains. ***, *P* < 0.001.

As SpoT is involved in the formation of the biofilm and multidrug resistance of H. pylori, along with an efflux pump (25, 33), we inferred that SpoT could possibly carry out those functions by regulating the expression of the efflux pump. Therefore, we compared the efflux activity of the WT, Δ*spoT*, and spoT* strains. The results revealed that the inactivation of SpoT, whether it was in biofilm-forming or planktonic cells, caused a distinct increase in the accumulation of Hoechst 33342, notably for planktonic cells, and the fluorescence values of the Δ*spoT* strain were >3-fold greater than those of the WT and spoT* strains. These results demonstrate that the efflux activity of the Δ*spoT* strain in response to Hoechst 33342 was weaker than that of the WT strain (Fig. 3), which suggests that SpoT likely regulates the efflux pump gene expression of H. pylori.

### Screening efflux pumps involved in biofilm formation and multidrug resistance in H. pylori by qRT-PCR.

**FIG 4:**
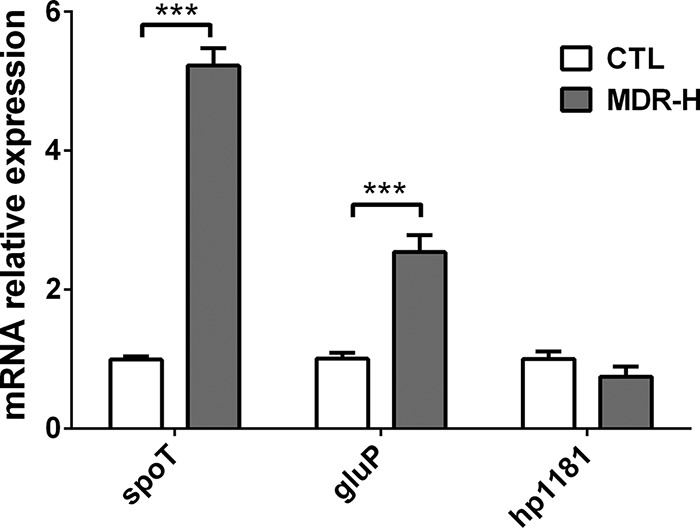
qRT-PCR analysis of the mRNA levels of *spoT*, *gluP* (Hp1174), and Hp1181 in the MDR-H strain (selected artificially) compared to those in the WT. The signals were normalized to the 16S rRNA levels. Data are the means ± standard errors of the means from three independent experiments. Significance was determined by the paired Student's *t* test. ***, *P* < 0.001. CTL, control.

We analyzed the difference in expression of some efflux pumps belonging to the major facilitator superfamily (MFS) and ATP-binding cassette (ABC) superfamily between biofilm-forming and planktonic cells by using quantitative real-time PCR (qRT-PCR) (Table 2). As seen in Table 2, the expression levels of two particular genes increased greater than 3-fold in biofilm-forming cells compared to those in to planktonic cells: Hp1181 (multidrug transporter) and Hp1174 (*gluP*). Furthermore, we analyzed the difference in mRNA expression of these two genes in an artificially selected multidrug-resistant H. pylori strain (MDR-H), and only *gluP* was highly expressed compared to its expression in the sensitive strain (H. pylori 26695) (Fig. 4). Considering that SpoT is highly expressed in MDR-H (Fig. 4) and biofilm-forming cells, we proposed that SpoT may participate in the biofilm formation and multidrug resistance of H. pylori by upregulating *gluP*.

**TABLE 2:** qRT-PCR analysis of efflux pump expression difference between biofilm-forming and planktonic H. pylori cells

Efflux pump family	Locus tag	Predicted function	Fold change in expression between biofilm and planktonic cells	*P* value for significance^*a*^
Major facilitator superfamily (MFS)	Hp0313	Nitrite	0.46 ± 0.14	*
	Hp0936	Proline/betaine	1.08 ± 0.17	
	Hp1091	Alpha-ketoglutarate	0.58 ± 0,03	**
	Hp1165	Multidrug efflux	2.66 ± 0.86	
	Hp1174	Glucose/galactose transporter	**5.75 ± 0.68**^*b*^	***
	Hp1181	Multidrug efflux	**3.34 ± 0.31**	***
	Hp1185	Sugar efflux	0.45 ± 0.27	*
ATP-binding cassette (ABC) superfamily	Hp1220	Multidrug efflux	2.99 ± 0.32	**
	Hp1082	Multidrug efflux	2.02 ± 0.25	
	Hp1486	Multidrug efflux	1.11 ± 0.29	
	Hp1206	Multidrug efflux	1.93 ± 0.17	**

a Significance was determined using the paired Student's *t* test. *, *P* < 0.05; **, *P* < 0.01; ***, *P* < 0.001.

b The boldface data indicate that the expression levels of the two genes increased greater than 3-fold in biofilm-forming cells compared to those in planktonic cells.

### SpoT regulates GluP expression.

**FIG 5:**
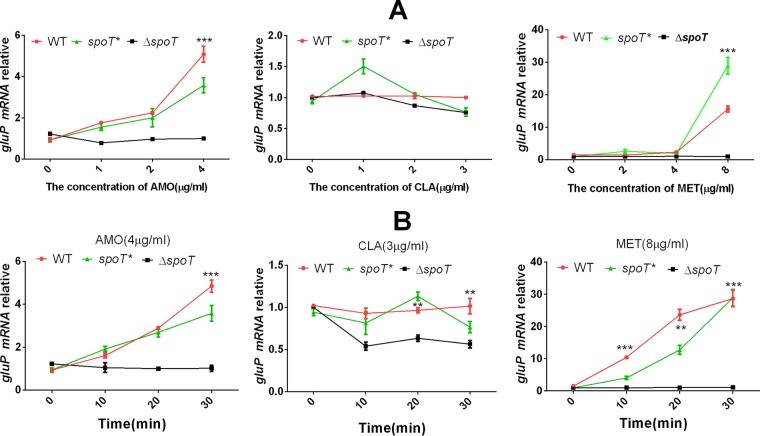
qRT-PCR analysis of the mRNA levels of *gluP* in the WT, Δ*spoT*, and spoT*strains. The WT, Δ*spoT*, and spoT*strains were exposed to different drug concentrations for 10 min (A) and exposed to specific drug concentrations for different time periods (B). The results were compared to those for the WT without drug treatment (control). The signal was normalized to the 16S rRNA levels. Data are the means ± standard errors of the means from three independent experiments. Significance was determined by the paired Student's *t* test between the WT and Δ*spoT* strains. **, *P* < 0.01; ***, *P* < 0.001.

To determine whether GluP is regulated by SpoT, we chose three antibiotics (CLA, AMO, and MET) with which to treat the WT, Δ*spoT*, and spoT* strains. First, we applied the three antibiotics at different concentrations to the WT, Δ*spoT*, and spoT* strains for 10 min; then, we applied a specific concentration of the three antibiotics along a time gradient. The qRT-PCR results showed that both AMO and MET could induce the expression of *gluP* in the WT and spoT* strains but not in the control group (the WT strain without antibiotic treatment), doing so in a concentration- and time-dependent manner, while in the Δ*spoT* strain, GluP could barely be induced by these two antibiotics (Fig. 5). However, CLA could hardly induce the expression of *gluP*, and the sensitivity of the WT and Δ*spoT* strains to CLA was similar according to the MIC data. This suggests that *gluP* may not be involved in the resistance of H. pylori to CLA. In short, the results presented above indicate that SpoT upregulates GluP to cope with specific antibiotic stress.

### GluP is involved in H. pylori efflux.

**FIG 6:**
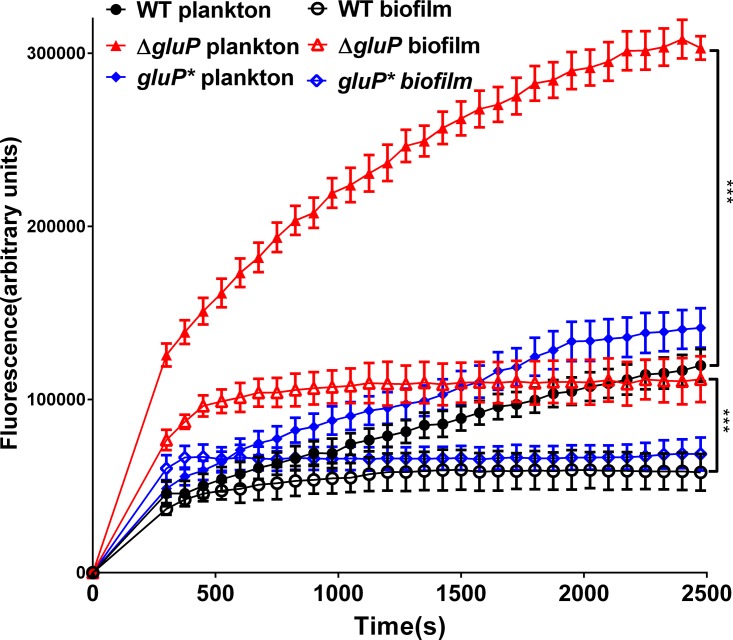
Comparison of the accumulation of Hoechst 33342 (2.5 M) in biofilm and planktonic cells of the WT, Δ*gluP*, and gluP* strains. The fluorescence intensity was recorded at excitation and emission wavelengths of 350 and 460 nm, respectively, over a 30-min incubation period. The data presented are the means ± standard errors of the means from three separate experiments. The paired Student's *t* test was performed to compare the accumulation of Hoechst 33342 between the WT and Δ*gluP* strains. ***, *P* < 0.001.

GluP, a glucose transporter, is responsible for glucose transport in H. pylori (21, 39). In addition, structural analysis demonstrates that GluP is an efflux pump belonging to the MFS, which suggests that GluP likely functions in drug efflux in H. pylori. Therefore, we successfully constructed a *gluP* mutant strain (the Δ*gluP* strain) and a *gluP*-complemented strain (the gluP* strain) and compared the efflux capacity of the WT, Δ*gluP*, and gluP* strains. These results revealed that the inactivation of GluP caused a distinct increase in the accumulation of Hoechst 33342, whether it was in biofilm-forming or planktonic cells but especially in planktonic cells, and the fluorescence values of the Δ*gluP* strain were >3-fold greater than those of the WT and gluP* strains. These results showed that the Δ*gluP* strain had a lower efflux capacity for Hoechst 33342 than the WT strain in both planktonic and biofilm-forming cells (Fig. 6).

### GluP is involved in H. pylori biofilm formation and multidrug resistance.

**FIG 7:**
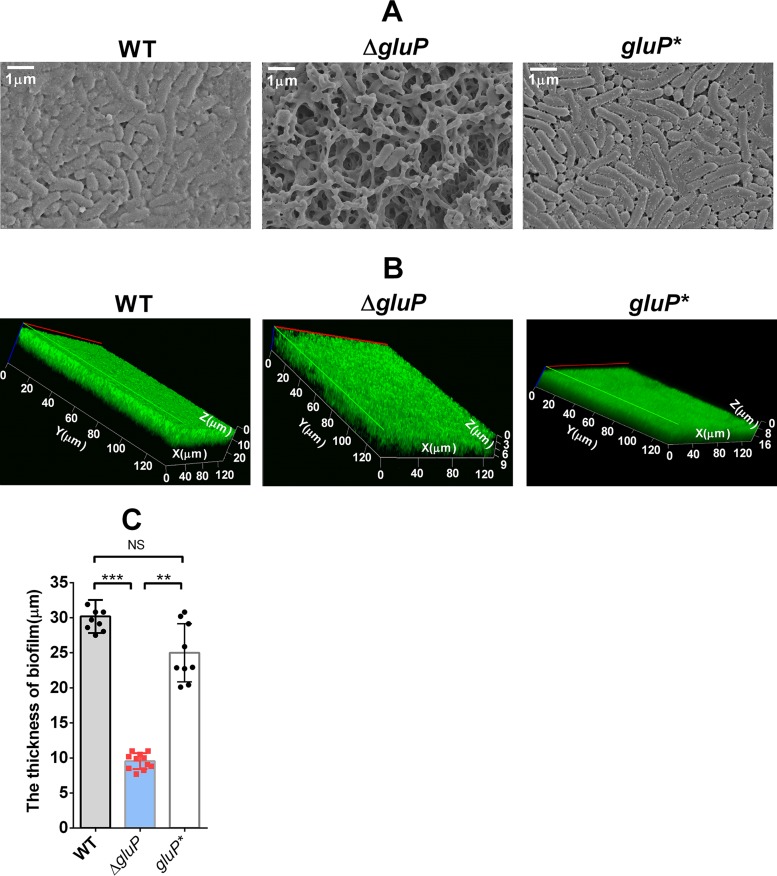
GluP is involved in H. pylori biofilm formation. (A) Scanning electron microscopy (SEM) images of WT, Δ*gluP*, and gluP* strain biofilms. (B) Confocal laser scanning microscopy (CLSM) images of WT, Δ*gluP*, and gluP* strain biofilms. Cells stained with membrane-permeant SYTO 9 (green) and membrane-impermeant propidium iodide (PI) (red) were visualized by confocal microscopy. (C) Comparison of the biofilm thickness between the WT, Δ*gluP*, and gluP* strains, using data from the assay whose results are presented in panel B. The data presented are the means ± standard errors of the means from three independent experiments. Significance was determined by the paired Student's *t* test. **, *P* < 0.01; ***, *P* < 0.001. The biofilm used in this experiment is a mature biofilm grown on a nitrocellulose membrane for 3 days, while the planktonic H. pylori bacteria were from the early exponential phase (OD_600_, approximately 0.4 to 0.5).

Studies have reported that efflux pumps participate in bacterial biofilm formation (33), so we compared the biofilms of the WT, Δ*gluP*, and gluP* strains by SEM. The images showed that compared with the bacteria in the biofilms of the WT and gluP* strains, the bacteria in the biofilm of the Δ*gluP* strain were not tightly packed, and the biofilm matrix was incomplete and showed more cavities (Fig. 7A). By CLSM, LIVE/DEAD cell viability assays showed that the Δ*gluP* strain formed a thinner biofilm (Fig. 7B and C).

According to the MIC for planktonic cells of the WT strain and the Δ*gluP* strain (Table 1), the Δ*gluP* strain was apparently more sensitive to various antibiotics than the WT strain, except for its sensitivity to CLA and ciprofloxacin. After knocking out *gluP* in the MDR-H strain, its resistance to drugs, especially its resistance to AMO, also decreased significantly (Table 1). In addition, for those biofilm-forming cells, after knocking out *gluP*, their sensitivity to various antibiotics increased (Table 1).

**FIG 8:**
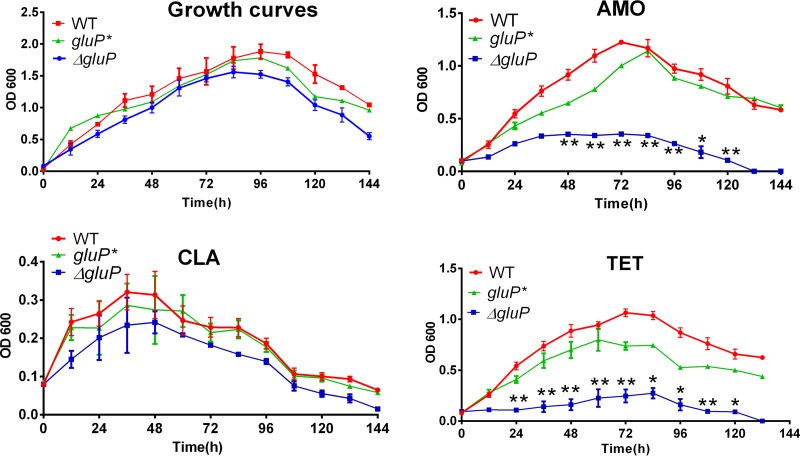
Comparison of growth curves and growth inhibition curve characteristics of the WT, Δ*gluP*, and gluP* strains. (A) Growth curves of the WT and Δ*gluP* strains. (B, C, and D) Growth inhibition curves of the WT, Δ*gluP*, and gluP* strains exposed to AMO (0.125 μg/ml), CLA (0.125 μg/ml), and MET (0.5 μg/ml). Data are the means ± standard errors of the means from three independent experiments. Significance was determined by the paired Student's *t* test. *, *P* < 0.05; **, *P* < 0.01.

We treated the WT, Δ*gluP*, and gluP* strains separately with three antibiotics (CLA, AMO, and MET) at the MIC (for the WT strain) and then observed their growth inhibition curves. The results showed that compared with the growth of the WT and gluP* strains, the growth of the Δ*gluP* strain was significantly inhibited by AMO and MET but not by CLA (Fig. 8). The results presented above suggest that GluP may not be involved in the tolerance of planktonic H. pylori bacteria to CLA, which is consistent with the results of MIC testing.

**FIG 9:**
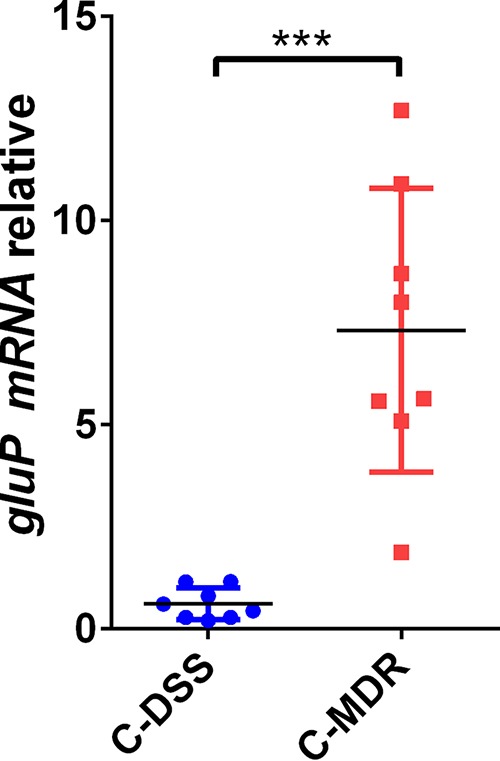
Analysis of *gluP* expression differences in clinical drug-sensitive strains (C-DSS) (*n* = 8) and clinical multidrug-resistant strains (C-MDR) (*n* = 8) by qRT-PCR. The results were compared to the value for *gluP* in the WT. The signal was normalized to the 16S rRNA levels. Data are the means ± standard errors of the means from three independent experiments. Significance was determined by the paired Student's *t* test. ***, *P* < 0.001.

The relative mRNA expression levels of *gluP* were assessed by qRT-PCR in the clinical MDR strains (C-MDR) and clinical drug-sensitive strains (C-DSS). The average expression levels of *gluP* in the C-MDR were significantly higher than those in the C-DSS (Fig. 9).

## DISCUSSION

Recently, the antibiotic resistance acquired by H. pylori has generally increased, and the formation of biofilms *in vivo* is an important cause leading to this multidrug resistance (10). In this research, we found that SpoT is involved in biofilm formation in H. pylori. While it has been reported that the efflux pump functions in the formation of biofilms and multidrug resistance (33), we found that *gluP* is involved in the biofilm formation and multidrug resistance of H. pylori. Further analysis showed that the expression of *gluP* is upregulated by SpoT. Considering that SpoT is a global regulator, our study partly explains the molecular mechanism by which SpoT is involved in the biofilm formation and multidrug resistance of H. pylori.

Antibiotics, as signaling molecules (40), can induce bacteria to produce (p)ppGpp and lead to a stringent response (14). Studies have shown that (p)ppGpp is involved in bacterial tolerance to various antibiotics, such as penicillin (41), vancomycin (42), and ampicillin (43); in H. pylori, (p)ppGpp is synthesized from SpoT (21). Our previous studies discovered that (p)ppGpp modulates the expression of H. pylori efflux transporters (44), accompanied by the importance of the efflux pump to multidrug resistance (24); thus, we wondered whether (p)ppGpp participates in the multidrug resistance of H. pylori. This research confirmed that inference.

Biofilm formation is another important factor that causes multidrug resistance in bacteria (7). Biofilms are communities of microorganisms anchored to a surface and live in an extracellular matrix that is made up of EPS produced by the organisms to form their immediate environment (7). Bacteria with biofilms have strong resistance to antibiotics and host immune defenses (6). It has been reported that (p)ppGpp is involved in the formation of biofilms in various bacteria, but the function of (p)ppGpp during this process varies from species to species. Several previous studies have shown that the lack of (p)ppGpp resulted in decreased biofilm formation in bacteria, such as Enterococcus faecalis (38), Vibrio cholerae (45), and Bordetella pertussis (16). However, other studies have proven that (p)ppGpp synthase deletion mutants of Actinobacillus pleuropneumoniae (17) and Francisella novicida (46) can produce significantly more biofilms than can the WT. Since *spoT* is the only gene that plays a part in the synthesis of (p)ppGpp in H. pylori, its ability to synthesize (p)ppGpp is totally lost after the knockout of *spoT*, consequently lowering its ability to adapt to stressful environments (19, 20, 22). In this study, we induced H. pylori to form a biofilm through nutrient starvation because nutrition deficiency is a significant factor that induces bacteria to form biofilms. After *spoT* knockout, the ability of H. pylori to form biofilms was greatly reduced.

Biofilm formation is an important cause of multidrug resistance in bacteria, and biofilms lead to continuous chronic infections, which add to the difficulty in providing a clinical cure (6). Drug resistance mechanisms include the following: poor diffusion of antibiotics through the biofilm polysaccharide matrix, physiological changes due to the low growth rate and starvation responses that form persistent cells, phenotypic changes of the cells forming the biofilm, and the expression of efflux pumps (7). Drug efflux is a key mechanism of drug resistance in Gram-negative bacteria (24). Microorganisms regulate the internal environment to adapt to their outer circumstances by excluding poisonous substances (antimicrobial agents, metabolites, and quorum-sensing chemical signals) via efflux pumps (25). There are six families of bacterial efflux pumps: (i) the ABC superfamily, (ii) the MFS, (iii) the multidrug and toxic compound extrusion (MATE) family, (iv) the small multidrug resistance (SMR) family, (v) the resistance-nodulation-division (RND) superfamily, and (vi) the drug metabolite transporter (DMT) superfamily (33). It is generally agreed that the ABC, MFS, and RND families play important roles in Gram-negative bacteria (33). As extensive studies regarding the RND family in H. pylori have been conducted (8, 28, 32, 37), we focused on the function of the MFS and the ABC superfamily.

Normally, the expression of efflux pumps in biofilm-forming bacteria is higher than that in planktonic cells (33). For example, in P. aeruginosa, the mechanism for tolerance to colistin is the upregulation of the MexAB-OprM efflux pump in biofilms (47). Moreover, in E. coli cells grown in biofilms and exposed to several antibiotics, the genes encoding the AcrAB-TolC efflux pump are upregulated (48). In addition, the expression levels of the *acrA* and *acrB* genes have been observed to increase in Salmonella biofilm cells (49). It has been reported that the RND family of efflux pumps is highly expressed in H. pylori biofilms (37), and recent studies have shown that Hp1165 and *hefA* are highly expressed in clinically isolated H. pylori biofilms (50). In our research, the expression of several efflux pumps was high in H. pylori biofilms. Some further studies of *gluP* found that efflux pump expression functioned in the formation of H. pylori biofilms. After *gluP* knockout, the H. pylori biofilm matrix is damaged and the bacteria are unable to form a well-structured biofilm. Some studies confirmed that the extremely low level of biofilm formation observed in the WT can be observed in E. coli mutants without *emrD*, *emrE*, *emrK*, *acrD*, *acrE*, and *mdtE* efflux pump-encoding genes (51). Efflux pumps are involved in biofilm formation, possibly due to the export or import of some substances related to biofilm formation. *gluP* is a glucose/galactose transporter belonging to the MFS (21), which is mainly responsible for the physiological uptake of sugars, such as d-glucose, into H. pylori (39). d-Glucose is involved in the synthesis of bacterial exopolysaccharides, while polysaccharides account for a major fraction of the biofilm matrix (52). Therefore, the deletion of *gluP* may affect the synthesis of the H. pylori biofilm matrix. We found that the matrix of the H. pylori biofilm was incomplete after *gluP* knockout, even if the concentration of glucose in the medium was increased (see Fig. S1 in the supplemental material). In addition, the biofilm matrix can limit the transport of some antimicrobial agents to cells within the biofilm (7).

The results of the growth inhibition curve (Fig. 8) and MIC (Table 1) analyses showed that knocking out *gluP* did not affect the sensitivity of H. pylori to CLA, suggesting that *gluP* may not be involved in the tolerance of H. pylori to CLA. In fact, although the efflux pump is an important weapon for multidrug resistance, it has a certain specificity for antibiotic substrates (53); for example, the AcrAB-TolC pump is not involved in telithromycin efflux in Enterobacter aerogenes or Escherichia coli (54). Our results suggest that CLA is not a good substrate for *gluP*.

Extensive studies concerning the regulatory mechanism of efflux pumps, such as two-component signal (TCS) transduction systems, local repressors, and global response regulators, have been conducted (55). In E. coli, (p)ppGpp can also regulate the expression of the efflux pump of YojI (56). There is one possible mechanism that explains how (p)ppGpp indirectly mediates global changes at the transcriptional level, such as by reducing the RpoD competitiveness for the core RNA polymerase. (p)ppGpp releases RpoD from the RNA polymerase and shifts the use of the alterative sigma factors (57). It is known that the H. pylori genome includes only two alterative sigma factors (σ^54^ and σ^28^) (21). According to the research of Niehus and coworkers, the promoter sequence of σ^54^-dependent genes is TTTGCTT (58). By analyzing the upstream sequence of the putative ATG start codon of the open reading frame of *gluP*, we discovered a possible conserved sequence (TTTGCAT) that was recognized by σ^54^ (Fig. S2), which suggests that *gluP* could be regulated by σ^54^. In further studies, antibiotic treatment could not induce high *gluP* expression in the σ^54^ mutant strain (Fig. S3). Therefore, SpoT may upregulate the expression of *gluP* by σ^54^-dependent transcription, but detailed mechanisms require more studies.

In conclusion, this research has discovered a new mechanism regarding biofilm formation and multidrug resistance in H. pylori. Further analysis is required to identify the specific mechanisms by which SpoT regulates H. pylori biofilm formation. On account of the present data, our research provides evidence and clues for the clinical treatment of patients infected with drug-resistant strains and epidemiological investigation.

## MATERIALS AND METHODS

### Bacterial strain, media, growth conditions, and clinical isolation of H. pylori.

H. pylori 26695, which was kindly provided by Zhang Jianzhong (Chinese Disease Control and Prevention Center), was used in this study. The strain was maintained at −80°C in brucella broth (BB; Difco, Detroit, MI) with 20% (vol/vol) glycerol and 10% fetal bovine serum (FBS). The strain was cultured under a microaerobic environment (5% O_2_, 10% CO_2_, 85% N_2_) at 37°C on brucella agar plates containing 7% lysed sheep blood. The liquid culture medium for H. pylori consisted of BB containing 10% FBS, and the cells were incubated in a shaker set at 120 rpm at 37°C. The mutant strains were cultured on agar plates with kanamycin (Sigma-Aldrich, St. Louis, MO) at 30 μg/ml. The E. coli strain was TOP10 (TransGen Biotech, Beijing, China) and was grown in Luria-Bertani medium at 37°C.

Eight MDR clinical isolates and eight sensitive clinical isolates were obtained from patients, including those with gastritis, gastric ulcers, duodenal ulcers, and gastric cancer, at Qiannan People's Hospital (Guizhou Province). All the patients provided informed consent before examination. The study was approved by the ethics committee of School of Medicine, Shandong University. MDR H. pylori strains from the patients could not be killed with repeated standard triple-therapy treatment. Isolated H. pylori strains were identified using universally accepted phenotypic tests: typical morphology on Gram-stained smears; and positive urease, oxidase, and catalase tests. The names of the C-DSS (*n* = 8) and C-MDR (*n* = 8) are listed in Table S1 in the supplemental material.

### Assessment of susceptibility to antibiotics.

The MICs of various antibiotics for all the clinical and standard strains were determined by the Etest and the agar dilution method as reported by Osato et al. (59). The bacteria (optical density at 600 nm [OD_600_], 0.8) were inoculated on an agar plate containing 2-fold dilutions of antibiotics. All the plates were incubated at 37°C under microaerobic conditions, and MIC values were determined. The method used to determine the MICs for biofilm-forming bacteria was similar to that used for the planktonic bacteria. The biofilm, which was attached to a nitrocellulose (NC) membrane, was incubated in a liquid medium containing different concentrations of various antibiotics for 12 h. After being washed three times with phosphate-buffered saline (PBS), the biofilm bacteria were suspended in liquid culture medium, and this liquid was inoculated on an agar plate to determine the MIC values.

### Construction of biofilm.

The biofilm could be cultivated under two kinds of conditions: the static condition or the continuous-flow condition. There are many ways to cultivate biofilms under the static condition; we used the colony biofilm (described in previous articles [60, 61]) with slight modification. To allow the adherence of H. pylori at the interface, 20 μl of bacteria was inoculated at 5 × 10^7^ cells ml^−1^ onto an NC membrane (approximately 1 by 1 cm^2^), which was placed on an agar plate. The agar plate was cultured in a microaerobic environment (5% O_2_, 10% CO_2_, 85% N_2_) at 37°C for 3 days.

### SEM.

To observe the H. pylori biofilm, scanning electron microscopy (SEM) was used. The samples for SEM were prepared using the following standard procedures. Planktonic bacteria were fixed with glutaraldehyde after centrifugation. For the biofilm-forming bacteria, the samples were gently washed with autoclaved PBS three times to remove the planktonic bacteria. The biofilms were fixed in 2.5% glutaraldehyde at 4°C for 2 h and then washed three times with cacodylate buffer and dehydrated through a series of graded ethanol solutions (25%, 50%, 75%, 95%, and 100%). Subsequently, the samples were freeze-dried, sputter coated with gold, and observed by SEM.

### CLSM.

To determine bacterial shape and viability, planktonic H. pylori bacteria were stained with membrane-permeant and membrane-impermeant fluorescent dyes from LIVE/DEAD BacLight bacterial viability kits (Molecular Probes, Invitrogen, USA). Then, they were observed by confocal microscopy, which was performed as described in a previous study (44). For biofilm-forming cells, the NC membrane with the biofilm was removed from the agar plate and then gently washed three times with PBS. Subsequently, the NC membrane was stained in a 12-well plate with 1 ml of SYTO 9 dye for 20 min under dark conditions and gently washed three times with PBS. After that, the NC membrane was fixed on a slide, covered with a coverslip, and subsequently analyzed by confocal laser scanning microscopy (CLSM; Leica TCS SP5; Leica Microsystems GmbH, Wetzlar, Germany). SYTO 9 is a green fluorescent membrane-permeant dye that labels all bacteria by staining nucleic acid, whereas propidium iodide (PI) is a red fluorescent membrane-impermeant dye that labels only bacteria with damaged membranes.

### Detection of (p)ppGpp accumulation patterns.

(p)ppGpp production was assayed according to the method described in a previous study with slight modification (44). The treatment of the planktonic bacteria was the same as previously described (44). The biofilm bacteria were washed with PBS, diluted to an OD_600_ of 0.2, and incubated for an additional 2 h. When all the strains reached an OD_600_ of approximately 0.3, samples from each culture plate were centrifuged at 10,000 rpm for 5 min and resuspended in 250 μl of liquid culture medium. ^32^P (Amersham) was added to 100 μCi ml^−1^, and the cultures were labeled for 2 h at 37°C. Then, 50 μl of each sample was added to an equal volume of 2 M formic acid. Afterward, at least four freeze-thaw cycles were conducted. The acid extracts were briefly centrifuged, and the supernatants were spotted onto polyethyleneimine-cellulose plates (Sigma-Aldrich), dried, and developed in 1.5 M KH_2_PO_4_ (pH 3.4) for approximately 2.5 h. The results were obtained under phosphor screen scanning (Bio-Rad).

### Extraction of RNA and quantitative real-time PCR (qRT-PCR).

Total RNA was extracted using the TRIzol reagent (Invitrogen, Carlsbad, CA). To reverse transcribe the total RNA, a PrimeScript RT reagent kit with gDNA Eraser (TaKaRa) was used. The primers used are shown in Table 3. The 20-μl quantitative PCR mixtures contained 5 μl of the resulting cDNA, which was already diluted; 10 μl of SYBR Premix Ex Taq (TaKaRa, Otsu, Shiga, Japan); 0.8 μl of the primer mixture; and 4.2 μl of double-distilled water. Then, RT-PCR was performed using an ABI Prism 7000 sequence detection system (Applied Biosystems, Carlsbad, CA) for 1 cycle at 95°C for 30 s and 40 cycles at 95°C for 5 s and 60°C for 31 s. Dissociation curve analysis was performed to verify the product homogeneity. The 16S rRNA amplicon was used as an internal control for data normalization. Changes in the transcript level were determined by applying the relative quantitative (ΔΔ*C_T_*) method. The threshold cycle (*C_T_*) values for all three biological replicates for each strain were compiled.

**TABLE 3:** Primers used in this study^*a*^

Forward primers		Reverse primers	
Name	Sequence (5′–3′)	Name	Sequence (5′–3′)
hp1174SF	CGGGATCCTATTATTGGGGAGGCGCGAT	hp1174SR	CCATCGATAGCTCTGTCCTCATCAAATACTG
hp1174XF	AACTGCAGACTTCTAACACTCTGGCGCT	hp1174XR	CGGAATTCCACCGCCAAGCCCAAATAAG
hp1174CF	CGGAATTCATGCAAAAAACTTCTAACAC	hp1174CR	CGGGATCCTTAGGAGTTTTCTTCTTGCT
hp0313	CGCATGCTTTTTACCCATTT	hp0313	AGAAAACACCACCCACGAAG
hp0936	ACGCGCCAAGTTTAGTCAAT	hp0936	CAGGCGCTGCTATAGCTTTT
hp1091	TGCGTTCTTAGCGCCTTATT	hp1091	GCGCCATAAGGATAATGGAA
hp1165	AGGGAGTTCTTTGGGATCGT	hp1165	AAGACGGGCGTAATCAAATG
hp1174	CCGCTGGTAATCCCTTTGTA	hp1174	CTTGCATTATCGCCCATTTT
hp1181	GGGGTGGCGTTTTTCTTTAT	hp1181	CCCCAAATAAGCCCTAAAGC
hp1185	ATCTCTGGGCATTTCACCAC	hp1185	CCATCGCAAAAGCGATAAAT
hp1220	CAAAAGGCATGAGGGAAAAA	hp1220	TTGCGTTTTGGCTAAATTCC
hp1082	TGCCGTTAGCTGCTATTCCT	hp1082	ACGGCGATGTTTTTGATACC
hp1486	AAATGAAGCCCACACCACTC	hp1486	TAAATTCCGCATGCATTTGA
hp1206	TTTTCCTGCTTGTGCTGATG	hp1206	CCCCACCAAGCAAAAACTAA
hp0714	GGGTTTTCCCCATTAAGCAT	hp0714	AGAGGCGATGTTGAGCAGTT

a Underlining indicates nucleotides that were added at the 5′ end to create a restriction site.

### Construction of Hp1174 (*gluP*) and *spoT* mutants and complemented strains.

In our previous studies, we successfully constructed an *spoT* mutant strain (the Δ*spoT* strain) (62) and an *spoT*-complemented strain (the spoT* strain) (44). The plasmids (pILL570 and pUC18K2) used to construct the mutant strain were kindly provided by Agnès Labigne (Unité de Pathogénie Bactérienne des Muqueuses, Institut Pasteur). The construction of the *gluP* mutant strain was identical to the construction of the Δ*spoT* strain, as described in a previous study (62). Briefly, the *gluP* gene from the genome of H. pylori 26695 was destroyed with the insertion of the nonpolar *aphA-3* gene, encoding a kanamycin resistance cassette. The construction of the *gluP*-complemented strain was similar to the construction of the spoT* strain. Briefly, the *gluP*-complemented strain (the gluP* strain) was constructed using the chloramphenicol resistance cassette from pMcagA, which was kindly presented by Wei Hong (Department of Microbiology, Guizhou Medical University, China). Full-length *gluP* was cloned into pMcagA, and the resulting plasmid was inserted into the middle of the Hp0547 (*cagA*) gene, which provided homologous recombination sites in H. pylori. The vector-transformed *gluP* mutant strain was constructed by electroporation to obtain the *gluP*-complemented strain (the gluP* strain). The genotype of the complemented gluP* transformant was verified by PCR and sequencing of the genomic loci. The primers used in these studies are listed in Table 3.

### Determination of growth curves and growth inhibition curves.

The growth curves were determined as described in a previous study (44). The growth profiles in BB with a preliminary OD_600_ of 0.08 were monitored, and then the bacteria were cultured for another 144 h at 37°C with shaking. Records were taken every 12 h by determining the OD_600_ of the test strain. The values stated are the mode values from at least three biological replicates performed on at least three independent occasions.

To analyze the growth inhibition curve, the H. pylori strains were inoculated into BB, which also contained different antibiotics (at the MIC for the WT strain), with a preliminary OD_600_ of 0.08, and then the bacteria were cultured for another 144 h at 37°C with shaking. Each experiment was repeated at least three times.

### Hoechst 33342 accumulation assay.

For the planktonic bacteria, the accumulation assay was performed as described previously (44). The biofilm bacteria were first rinsed with PBS and subsequently suspended in PBS, with the final OD_600_ being 0.1. Then, 180 μl of this liquid and 20 μl of Hoechst 33342 (25 μM; Sigma-Aldrich) were added to each well of a 96-well plate. Recording began 5 min after the addition of Hoechst 33342. Excitation and emission were measured at 355 nm and 460 nm, respectively, using a FLUOstar Optima microplate reader (BMG LABTECH, Aylesbury, UK). Readings were taken every 75 s for 30 cycles, and the raw data were analyzed by use of the Excel program. Each experiment was repeated at least three times.

### Generation of MDR-H.

In order to obtain artificially selected multidrug-resistant H. pylori (MDR-H), H. pylori 26695 was cultivated on an agar plate containing 0.5× MIC of chloramphenicol for 48 to 72 h under a microaerobic environment at 37°C, as described in a previous study (63). The resistant colonies were incubated with repeated doubling of the chloramphenicol concentration until no colony was seen. Colonies were maintained on agar plates containing 4× MICs of TET, ampicillin, penicillin G, and erythromycin. Then, the colonies were incubated for 48 to 72 h under a microaerobic environment.

### Statistical analysis.

Data are presented as the means ± standard errors of the means. Statistical significance was determined using the unpaired Student's *t* test, and the *P* values were corrected by the Sidak-Bonferroni method for multiple comparisons. *P* values of <0.05 were considered statistically significant. The results were analyzed using GraphPad Prism software (GraphPad Software Inc., La Jolla, CA, USA).

## Supplementary Material

Supplemental file 1

## References

[1] Kusters JG, van Vliet AHM, Kuipers EJ 2006 Pathogenesis of *Helicobacter pylori* infection. Clin Microbiol Rev 19:449–490. doi:10.1128/CMR.00054-05. 16847081 PMC1539101

[2] Mégraud F 2004 H pylori antibiotic resistance: prevalence, importance, and advances in testing. Gut 53:1374–1384. doi:10.1136/gut.2003.022111. 15306603 PMC1774187

[3] Kuo Y-T, Liou J-M, El-Omar EM, Wu J-Y, Leow AHR, Goh KL, Das R, Lu H, Lin J-T, Tu Y-K, Yamaoka Y, Wu M-S 2017 Primary antibiotic resistance in *Helicobacter pylori* in the Asia-Pacific region: a systematic review and meta-analysis. Lancet Gastroenterol Hepatol 2:707–715. doi:10.1016/S2468-1253(17)30219-4. 28781119

[4] Kwon DH, Dore MP, Kim JJ, Kato M, Lee M, Wu JY, Graham DY 2003 High-level beta-lactam resistance associated with acquired multidrug resistance in *Helicobacter pylori*. Antimicrob Agents Chemother 47:2169–2178. doi:10.1128/AAC.47.7.2169-2178.2003. 12821464 PMC161855

[5] Blair JMA, Webber MA, Baylay AJ, Ogbolu DO, Piddock LJV 2015 Molecular mechanisms of antibiotic resistance. Nat Rev Microbiol 13:42–51. doi:10.1038/nrmicro3380. 25435309

[6] Flemming H-C, Wingender J, Szewzyk U, Steinberg P, Rice SA, Kjelleberg S 2016 Biofilms: an emergent form of bacterial life. Nat Rev Microbiol 14:563–575. doi:10.1038/nrmicro.2016.94. 27510863

[7] Stewart PS, Costerton JW 2001 Antibiotic resistance of bacteria in biofilms. Lancet 358:135–138. doi:10.1016/S0140-6736(01)05321-1. 11463434

[8] Stark RM, Gerwig GJ, Pitman RS, Potts LF, Williams NA, Greenman J, Weinzweig IP, Hirst TR, Millar MR 1999 Biofilm formation by *Helicobacter pylori*. Lett Appl Microbiol 28:121–126. doi:10.1046/j.1365-2672.1999.00481.x. 10063642

[9] Carron MA, Tran VR, Sugawa C, Coticchia JM 2006 Identification of *Helicobacter pylori* biofilms in human gastric mucosa. J Gastrointest Surg 10:712–717. doi:10.1016/j.gassur.2005.10.019. 16713544

[10] Yonezawa H, Osaki T, Kamiya S 2015 Biofilm formation by and its involvement for antibiotic resistance. Biomed Res Int 2015:914791. doi:10.1155/2015/914791. 26078970 PMC4452508

[11] Fruci M, Poole K 2016 Bacterial stress responses as determinants of antimicrobial resistance, p 115–136. *In* de Bruijn FJ (ed), Stress and environmental regulation of gene expression and adaptation in bacteria. John Wiley & Sons, Inc, Hoboken, NJ.

[12] Potrykus K, Cashel M 2008 (p)ppGpp: still magical? Annu Rev Microbiol 62:35–51. doi:10.1146/annurev.micro.62.081307.162903. 18454629

[13] Srivatsan A, Wang JD 2008 Control of bacterial transcription, translation and replication by (p)ppGpp. Curr Opin Microbiol 11:100–105. doi:10.1016/j.mib.2008.02.001. 18359660

[14] Wu J, Long Q, Xie J 2010 (p)ppGpp and drug resistance. J Cell Physiol 224:300–304. doi:10.1002/jcp.22158. 20432457

[15] Maisonneuve E, Castro-Camargo M, Gerdes K 2013 (p)ppGpp controls bacterial persistence by stochastic induction of toxin-antitoxin activity. Cell 154:1140–1150. doi:10.1016/j.cell.2013.07.048. 23993101

[16] Sugisaki K, Hanawa T, Yonezawa H, Osaki T, Fukutomi T, Kawakami H, Yamamoto T, Kamiya S 2013 Role of (p)ppGpp in biofilm formation and expression of filamentous structures in *Bordetella pertussis*. Microbiology 159:1379–1389. doi:10.1099/mic.0.066597-0. 23676431

[17] Li G, Xie F, Zhang Y, Bossé JT, Langford PR, Wang C 2015 Role of (p)ppGpp in viability and biofilm formation of *Actinobacillus pleuropneumoniae* S8. PLoS One 10:e0141501. doi:10.1371/journal.pone.0141501. 26509499 PMC4624843

[18] Atkinson GC, Tenson T, Hauryliuk V 2011 The RelA/SpoT homolog (RSH) superfamily: distribution and functional evolution of ppGpp synthetases and hydrolases across the tree of life. PLoS One 6:e23479. doi:10.1371/journal.pone.0023479. 21858139 PMC3153485

[19] Mouery K, Rader BA, Gaynor EC, Guillemin K 2006 The stringent response is required for *Helicobacter pylori* survival of stationary phase, exposure to acid, and aerobic shock. J Bacteriol 188:5494–5500. doi:10.1128/JB.00366-06. 16855239 PMC1540029

[20] Wells DH, Gaynor EC 2006 *Helicobacter pylori* initiates the stringent response upon nutrient and pH downshift. J Bacteriol 188:1–5. doi:10.1128/JB.188.1.1-18.2006. PMC1482847 16672627

[21] Tomb JF, White O, Kerlavage AR, Clayton RA, Sutton GG, Fleischmann RD, Ketchum KA, Klenk HP, Gill S, Dougherty BA, Nelson K, Quackenbush J, Zhou L, Kirkness EF, Peterson S, Loftus B, Richardson D, Dodson R, Khalak HG, Glodek A, McKenney K, Fitzegerald LM, Lee N, Adams MD, Hickey EK, Berg DE, Gocayne JD, Utterback TR, Peterson JD, Kelley JM, Cotton MD, Weidman JM, Fujii C, Bowman C, Watthey L, Wallin E, Hayes WS, Borodovsky M, Karp PD, Smith HO, Fraser CM, Venter JC 1997 The complete genome sequence of the gastric pathogen *Helicobacter pylori*. Nature 388:539–547. doi:10.1038/41483. 9252185

[22] Zhou YN, Coleman WG, Yang Z, Yang Y, Hodgson N, Chen F, Jin DJ 2008 Regulation of cell growth during serum starvation and bacterial survival in macrophages by the bifunctional enzyme SpoT in *Helicobacter pylori*. J Bacteriol 190:8025–8032. doi:10.1128/JB.01134-08. 18835987 PMC2593245

[23] Maloney PC 1994 Bacterial transporters. Curr Opin Cell Biol 6:571–582. 7986535 10.1016/0955-0674(94)90079-5

[24] Nikaido H 1996 Multidrug efflux pumps of gram-negative bacteria. J Bacteriol 178:5853–5859. doi:10.1128/jb.178.20.5853-5859.1996. 8830678 PMC178438

[25] Webber MA, Piddock LJV 2003 The importance of efflux pumps in bacterial antibiotic resistance. J Antimicrob Chemother 51:9–11. doi:10.1093/jac/dkg050. 12493781

[26] Bina JE, Alm RA, Uria-Nickelsen M, Thomas SR, Trust TJ, Hancock REW 2000 *Helicobacter pylori* uptake and efflux: basis for intrinsic susceptibility to antibiotics in vitro. Antimicrob Agents Chemother 44:248–254. doi:10.1128/AAC.44.2.248-254.2000. 10639345 PMC89666

[27] Van Amsterdam K, Bart A, Van Der Ende A 2005 A *Helicobacter pylori* TolC efflux pump confers resistance to metronidazole. Antimicrob Agents Chemother 49:1477–1482. doi:10.1128/AAC.49.4.1477-1482.2005. 15793129 PMC1068630

[28] Liu ZQ, Zheng PY, Yang PC 2008 Efflux pump gene hefA of *Helicobacter pylori* plays an important role in multidrug resistance. World J Gastroenterol 14:5217–5222. doi:10.3748/wjg.14.5217. 18777600 PMC2744013

[29] Hirata K, Suzuki H, Nishizawa T, Tsugawa H, Muraoka H, Saito Y, Matsuzaki J, Hibi T 2010 Contribution of efflux pumps to clarithromycin resistance in *Helicobacter pylori*. J Gastroenterol Hepatol 25(Suppl 1):S75–S79. doi:10.1111/j.1440-1746.2009.06220.x. 20586871

[30] Trainor EA, Horton KE, Savage PB, Testerman TL, McGee DJ 2011 Role of the HefC efflux pump in *Helicobacter pylori* cholesterol-dependent resistance to ceragenins and bile salts. Infect Immun 79:88–97. doi:10.1128/IAI.00974-09. 20974830 PMC3019907

[31] Tsugawa H, Suzuki H, Muraoka H, Ikeda F, Hirata K, Matsuzaki J, Saito Y, Hibi T 2011 Enhanced bacterial efflux system is the first step to the development of metronidazole resistance in *Helicobacter pylori*. Biochem Biophys Res Commun 404:656–660. doi:10.1016/j.bbrc.2010.12.034. 21147064

[32] Mehrabadi JF, Sirous M, Daryani NE, Eshraghi S 2011 Assessing the role of the RND efflux pump in metronidazole resistance of *Helicobacter pylori* by RT-PCR assay. J Infect Dev Ctries 5:88–93. doi:10.3855/jidc.1187. 21389587

[33] Soto SM 2013 Role of efflux pumps in the antibiotic resistance of bacteria embedded in a biofilm. Virulence 4:223–229. doi:10.4161/viru.23724. 23380871 PMC3711980

[34] De Kievit TR, Parkins MD, Gillis RJ, Srikumar R, Ceri H, Poole K, Iglewski BH, Storey DG 2001 Multidrug efflux pumps: expression patterns and contribution to antibiotic resistance in *Pseudomonas aeruginosa* biofilms. Antimicrob Agents Chemother 45:1761–1770. doi:10.1128/AAC.45.6.1761-1770.2001. 11353623 PMC90543

[35] Yamasaki S, Wang LY, Hirata T, Hayashi-Nishino M, Nishino K 2015 Multidrug efflux pumps contribute to *Escherichia coli* biofilm maintenance. Int J Antimicrob Agents 45:439–441. doi:10.1016/j.ijantimicag.2014.12.005. 25637119

[36] Ramage G 2002 Investigation of multidrug efflux pumps in relation to fluconazole resistance in *Candida albicans* biofilms. J Antimicrob Chemother 49:973–980. doi:10.1093/jac/dkf049. 12039889

[37] Yonezawa H, Osaki T, Hanawa T, Kurata S, Ochiai K, Kamiya S 2013 Impact of *Helicobacter pylori* biofilm formation on clarithromycin susceptibility and generation of resistance mutations. PLoS One 8:e73301. doi:10.1371/journal.pone.0073301. 24039906 PMC3765302

[38] Chávez de Paz LE, Lemos JA, Wickström C, Sedgley CM 2012 Role of (p)ppGpp in biofilm formation by *Enterococcus faecalis*. Appl Environ Microbiol 78:1627–1630. doi:10.1128/AEM.07036-11. 22179256 PMC3294496

[39] Psakis G, Saidijam M, Shibayama K, Polaczek J, Bettaney KE, Baldwin JM, Baldwin SA, Hope R, Essen LO, Essenberg RC, Henderson PJF 2009 The sodium-dependent d-glucose transport protein of *Helicobacter pylori*. Mol Microbiol 71:391–403. doi:10.1111/j.1365-2958.2008.06535.x. 19161491

[40] Yim G, Wang HH, Davies J 2007 Antibiotics as signalling molecules. Philos Trans R Soc Lond B Biol Sci 362:1195–1200. doi:10.1098/rstb.2007.2044. 17360275 PMC2435582

[41] Rodionov DG, Ishiguro EE 1995 Direct correlation between overproduction of guanosine 3′,5′-bispyrophosphate (ppGpp) and penicillin tolerance in *Escherichia coli*. J Bacteriol 177:4224–4229. doi:10.1128/jb.177.15.4224-4229.1995. 7635809 PMC177166

[42] Abranches J, Martinez AR, Kajfasz JK, Chávez V, Garsin DA, Lemos JA 2009 The molecular alarmone (p)ppGpp mediates stress responses, vancomycin tolerance, and virulence in *Enterococcus faecalis*. J Bacteriol 191:2248–2256. doi:10.1128/JB.01726-08. 19168608 PMC2655485

[43] Geiger T, Kästle B, Gratani FL, Goerke C, Wolz C 2014 Two small (p)ppGpp synthases in *Staphylococcus aureus* mediate tolerance against cell envelope stress conditions. J Bacteriol 196:894–902. doi:10.1128/JB.01201-13. 24336937 PMC3911181

[44] Geng X, Li W, Chen Z, Gao S, Hong W, Ge X, Hou G, Hu Z, Zhou Y, Zeng B, Li W, Jia J, Sun Y 2017 The bifunctional enzyme SpoT is involved in the clarithromycin tolerance of *Helicobacter pylori* by upregulating the transporters HP0939, HP1017, HP0497, and HP0471. Antimicrob Agents Chemother 61:e02011-16. doi:10.1128/AAC.02011-16. 28242673 PMC5404559

[45] He H, Cooper JN, Mishra A, Raskin DM 2012 Stringent response regulation of biofilm formation in *Vibrio cholerae*. J Bacteriol 194:2962–2972. doi:10.1128/JB.00014-12. 22467780 PMC3370634

[46] Dean SN, Chung MC, van Hoek ML 2015 *Burkholderia* diffusible signal factor signals to *Francisella novicida* to disperse biofilm and increase siderophore production. Appl Environ Microbiol 81:7057–7066. doi:10.1128/AEM.02165-15. 26231649 PMC4579433

[47] Pamp SJ, Gjermansen M, Johansen HK, Tolker-Nielsen T 2008 Tolerance to the antimicrobial peptide colistin in *Pseudomonas aeruginosa* biofilms is linked to metabolically active cells, and depends on the pmr and mexAB-oprM genes. Mol Microbiol 68:223–240. doi:10.1111/j.1365-2958.2008.06152.x. 18312276

[48] Bailey AM, Webber MA, Piddock LJV 2006 Medium plays a role in determining expression of *acrB*, *marA*, and *soxS* in *Escherichia coli*. Antimicrob Agents Chemother 50:1071–1074. doi:10.1128/AAC.50.3.1071-1074.2006. 16495271 PMC1426439

[49] Eaves DJ, Ricci V, Piddock LJV 2004 Expression of *acrB*, *acrF*, *acrD*, *marA*, and *soxS* in *Salmonella enterica* serovar Typhimurium: role in multiple antibiotic resistance. Antimicrob Agent Chemother 48:1145–1150. doi:10.1128/AAC.48.4.1145-1150.2004. PMC375282 15047514

[50] Attaran B, Falsafi T, Ghorbanmehr N 2017 Effect of biofilm formation by clinical isolates of *Helicobacter pylori* on the efflux-mediated resistance to commonly used antibiotics. World J Gastroenterol 23:1163–1170. doi:10.3748/wjg.v23.i7.1163. 28275296 PMC5323441

[51] Matsumura K, Furukawa S, Ogihara H, Morinaga Y 2011 Roles of multidrug efflux pumps on the biofilm formation of *Escherichia coli* K-12. Biocontrol Sci 16:69–72. doi:10.4265/bio.16.69. 21719992

[52] Limoli DH, Jones CJ, Wozniak DJ, Cruz S 2015 Bacterial extracellular polysaccharides in biofilm formation and function. Microbiol Spectr 3(3):MB-0011-2014. doi:10.1128/microbiolspec.MB-0011-2014. PMC4657554 26185074

[53] Van Bambeke F, Balzi E, Tulkens M 2000 Antibiotic efflux pumps. Biochem Pharmacol 60:457–470. doi:10.1016/S0006-2952(00)00291-4. 10874120

[54] Chollet R, Chevalier J, Bryskier A, Pagès JM 2004 The AcrAB-TolC pump is involved in macrolide resistance but not in telithromycin efflux in *Enterobacter aerogenes* and *Escherichia coli*. Antimicrob Agents Chemother 48:3621–3624. doi:10.1128/AAC.48.9.3621-3624.2004. 15328143 PMC514773

[55] Sun J, Deng Z, Yan A 2014 Bacterial multidrug efflux pumps: mechanisms, physiology and pharmacological exploitations. Biochem Biophys Res Commun 453:254–267. doi:10.1016/j.bbrc.2014.05.090. 24878531

[56] Pomares MF, Vincent PA, Farías RN, Salomón RA 2008 Protective action of ppGpp in microcin J25-sensitive strains. J Bacteriol 190:4328–4334. doi:10.1128/JB.00183-08. 18408024 PMC2446768

[57] Jishage M, Kvint K, Shingler V, Nyström T 2002 Regulation of σ factor competition by the alarmone ppGpp. Genes Dev 16:1260–1270. doi:10.1101/gad.227902. 12023304 PMC186289

[58] Niehus E, Gressmann H, Ye F, Schlapbach R, Dehio M, Dehio C, Stack A, Meyer TF, Suerbaum S, Josenhans C 2004 Genome-wide analysis of transcriptional hierarchy and feedback regulation in the flagellar system of *Helicobacter pylori*. Mol Microbiol 52:947–961. doi:10.1111/j.1365-2958.2004.04006.x. 15130117

[59] Osato MS, Reddy R, Reddy SG, Penland RL, Graham DY 2001 Comparison of the Etest and the NCCLS-approved agar dilution method to detect metronidazole and clarithromycin resistant *Helicobacter pylori*. Int J Antimicrob Agents 17:39–44. doi:10.1016/S0924-8579(00)00320-4. 11137647

[60] Franklin MJ, Bothner B, Akiyama T, Chang C 2015 New technologies for studying biofilms. Microbiol Spectr 3(4):MB-0016-2014. doi:10.1128/microbiolspec.MB-0016-2014. PMC4821632 26350329

[61] Merritt JH, Kadouri DE, O'Toole GA 2005 Growing and analyzing static biofilms. Curr Protoc Microbiol Chapter 1:Unit 1B.1. doi:10.1002/9780471729259.mc01b01s00. PMC4568995 18770545

[62] Sun Y, Li X, Li W, Zhao M, Wang L, Liu S, Zeng J, Liu Z, Jia J 2012 Proteomic analysis of the function of spot in *Helicobacter pylori* anti-oxidative stress in vitro and colonization in vivo. J Cell Biochem 113:3393–3402. doi:10.1002/jcb.24215. 22678710

[63] Zhang Z 2010 Influence of efflux pump inhibitors on the multidrug resistance of *Helicobacter pylori*. World J Gastroenterol 16:1279. doi:10.3748/wjg.v16.i10.1279. 20222174 PMC2839183

