# Supplementary material for: Bifunctional Enzyme SpoT Is Involved in Biofilm Formation of Helicobacter pylori with Multidrug Resistance by Upregulating Efflux Pump Hp1174 (gluP)
Source: Antimicrob Agents Chemother. 2018 Oct 24;62(11):e00957-18. doi: 10.1128/AAC.00957-18 (PMC6201075; doi:10.1128/AAC.00957-18)
Supplement: Supplemental file 1 [file zac011187608s1.pdf]

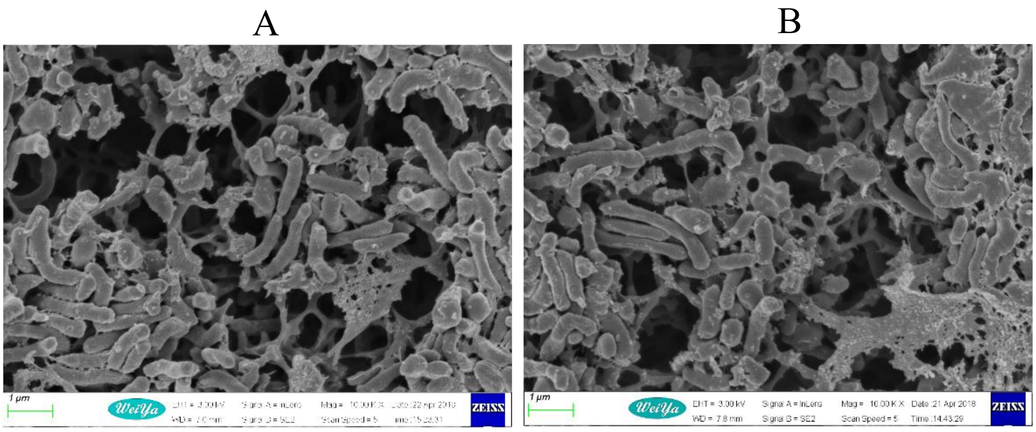

Fig. S1: Representative scanning electron microscopy (SEM) images of Δ*gluP* strain biofilms cultured on NC membranes with 1 g/L(A) and 4 g/L(B) glucose in an agar plate.

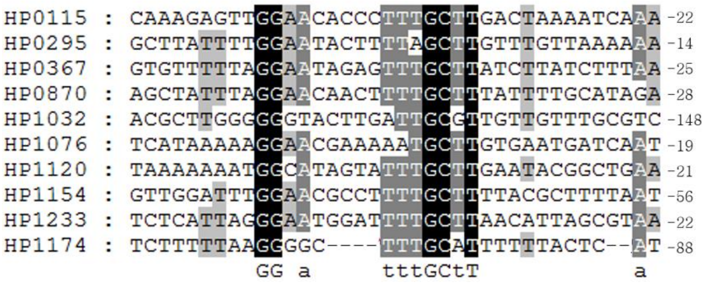

Fig. S2 Alignment of promoter sequences of four known σ^{54} -dependent genes [HP0115 (flaB), HP0295 (flgL), HP0870 (flgE1) and HP1120] , six genes and operons in *H. pylori* that belong to the RpoN regulon (Niehus et al., 2004), and *Hp1174*(*gluP*). Numbers to the right of the sequences indicate the distance from the putative ATG start codon of the ORFs. The bottom lane shows the derived consensus sequence. The shading was done using the GENEDOC software (http://iubio.bio.indiana.edu/soft/molbio/ibmpc/genedoc-readme.html). Black boxes indicate 100% conserved residues in all sequences; grey boxes indicate nucleotides conserved in more than half the promoters.

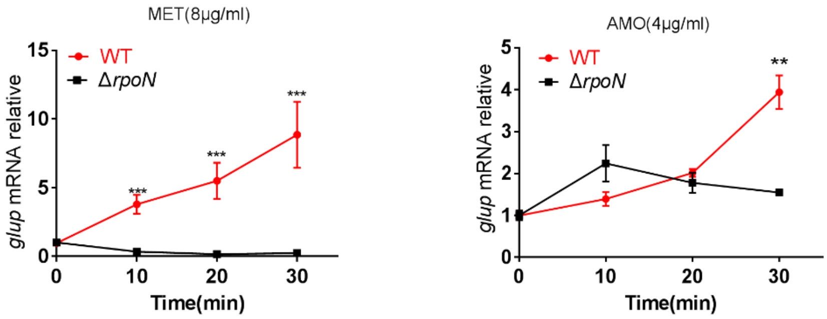

Fig. S3 qRT-PCR analysis of the mRNA levels of *gluP* in WT and Δ*rpoN* ($\Delta\sigma^{54}$) strains exposed to metronidazole(MET) and amoxicillin(AMO) for different time periods. The results were compared to WT without drugs treatment (CTL). The signal was normalized to the 16S rRNA levels. Data are means ±SEM from three independent experiments. Significance by paired *t* test: **, $P < 0.01$; ***, $P < 0.001$.

Table S1: The names of C-MDR and C-DSS strains used in this study. R:resistance, S:sensitive.

**Bacteria names**		**Antibiotics**				
		Clarithromycin	Amoxicillin	Levofloxacin	Tetracycline	Metronidazole
**C-MDR**	G295	R	S	R	S	R
	G236	R	S	R	S	R
	G141	R	S	R	S	R
	G28	R	S	S	S	R
	G124	R	S	R	S	S
	G450	R	S	R	S	R
	E660	S	R	S	R	S
	E325	R	S	R	R	R
**C-DSS**	E412	Sensitive				
	E702					
	G405					
	E313					
	E204					
	E222					
	G303					
	G352					

References

Niehus E, Gressmann H, Ye F, Schlapbach R, Dehio M, Dehio C, Stack A, Meyer TF, Suerbaum S, Josenhans C. 2004. Genome-wide analysis of transcriptional hierarchy and feedback regulation in the flagellar system of *Helicobacter pylori*. Mol Microbiol 52:947–961.
